# Prognostic Value and Immune Landscapes of m5C-Related lncRNAs in Lung Squamous Cell Carcinoma

**DOI:** 10.3389/fgene.2022.960229

**Published:** 2022-07-22

**Authors:** Ruoxin Xu, Wenxiong Zhang

**Affiliations:** ^1^ Department of Cardio-Thoracic Surgery, The Second Affiliated Hospital of Nanchang University, Nanchang, China; ^2^ Jiangxi Medical College, Nanchang University, Nanchang, China

**Keywords:** m5C, lncRNAs, immune, lung squamous cell carcinoma, prognosis

## Abstract

5-methylcytosine (m5C) modification is involved in tumor progression. However, the lncRNAs associated with m5C in lung squamous cell carcinoma (LUSC) have not been elucidated. The Cancer Genome Atlas database was used to get the open-accessed transcriptional profiling and clinical information of LUSC patients. All the statistical analyses were performed based on R software v 4.0.0 and SPSS13.0. First, there were 614 m5C-related lncRNAs identified under the criterion of |R|>0.4 and *p* < 0.001 with m5C genes. Next, a prognosis model based on ERICD, AL021068.1, LINC01341, AC254562.3, and AP002360.1 was established, which showed good prediction efficiency in both the training and validation cohorts. Next, a nomogram plot was established by combining the risk score and clinical features for a better application in clinical settings. Pathway enrichment analysis showed that the pathways of angiogenesis, TGF-β signaling, IL6-JAK-STAT3 signaling, protein secretion, androgen response, interferon-α response, and unfolded protein response were significantly enriched in the high-risk patients. Immune infiltration analysis showed that the risk score was positively correlated with neutrophils, resting CD4+ memory T cells, and M2 macrophages, yet negatively correlated with follicular helper T cells, CD8+ T cells, and activated NK cells. Moreover, we found that high-risk patients might be more sensitive to immunotherapy, imatinib, yet resistant to erlotinib, gefitinib, and vinorelbine. In summary, our prognosis model is an effective tool that could robustly predict LUSC patient prognosis, which had the potential for clinical guidance.

## Introduction

Worldwide, lung cancer has caused 2.2 million new cases and 1.8 million deaths in 2020, making it the leading cause of death from cancer (Sung et al., 2021). Among these, lung adenocarcinomas (LUAD) and squamous cell carcinomas (LUSC) are the two predominant pathological subtypes (Nooreldeen and Bach, 2021). Locally, advanced lung cancer is still treated with surgery as the preferred treatment option. However, a considerable proportion of patients suffer from disease recurrence and distant metastasis, resulting in poor prognosis (Yan et al., 2017). Therefore, identifying novel biomarkers of lung cancer might contribute to the diagnosis and treatment of lung cancer patients.

Epigenetic modifications, including DNA and RNA methylation, genomic imprinting, gene silencing, and non-coding RNA modification, can affect gene expression without changing the DNA sequence, which exerts an important role in tumor development (Kanwal et al., 2015). N6-methyladenine (m6A) has been proven to be widely involved in the progression process of the tumor. Researchers have observed RNA m5C modifications at mRNA and non-coding RNA along with the advances in high-throughput sequencing (Hu et al., 2021). For instance, Mei et al. found that through repression of p57 Kip2, NSUN2 could facilitate gastric cancer growth, which is m5C-dependent (Mei et al., 2020). Sun et al. (2020) indicated that the aberrant NSUN2-mediated m5C modification of H19 lncRNA is associated with poor differentiation of hepatocellular carcinoma. Mostly, m5C modification was catalyzed by methyltransferase complex, comprising three enzymes, including methyltransferase “Writer”, demethylase “Erase”, and m5C binding protein “Reader” (Chellamuthu and Gray, 2020). Meanwhile, m5C modification sites are also widely distributed in long non-coding RNAs (lncRNAs) (Squires et al., 2012). Previous studies have investigated the specific lncRNAs in cancers. Liu et al. (2021) developed a novel immune-related lncRNA signature, which might be a prognostic classifier for endometrial carcinoma. In liver cancer, Pan et al. (2022) identified 13 m5C-related lncRNAs, which are significantly associated with patient prognosis. However, there is little research focused on the relationship between lncRNA and m5C modification in LUSC.

In this study, we identified m5C-related lncRNAs based on the data downloaded from The Cancer Genome Atlas (TCGA)-LUSC project. Finally, five m5C-related lncRNAs such as ERICD, AL021068.1, LINC01341, AC254562.3, and AP002360.1 were identified for model construction, which demonstrated good prediction efficiency in both the training and validation cohorts. Next, pathway enrichment and immune infiltration analyses were performed to explore the underlying biological difference between high- and low-risk patients. Moreover, we found that high-risk patients might be more sensitive to immunotherapy, imatinib, yet resistant to erlotinib, gefitinib, and vinorelbine.

## Methods

### Public Data Acquisition

The transcriptome profile and clinical information of LUSC patients were obtained from the TCGA database (https://www.cancer.gov/tcga, accessed in 2022-06-02), which was collated using the author’s code. First, a total of 501 patients have the clinical information and expression profile data and therefore enrolled in our study. Six patients were then removed for their unknown survival time. The Homo_sapiens.GRCh38.106.chr.gtf reference file downloaded from the Ensembl website (http://asia.ensembl.org/index.html) was used for gene annotation. The tumor mutation burden (TMB) and microsatellite instability (MSI) were obtained from the TCGA database. Tumor stemness (mRNAsi) was calculated based on the expression profile using the one-class logistic regression (OCLR) machine learning algorithm (Malta et al., 2018). Before analysis, all the data were standardized and processed, including probe annotation, missing value completion, and data standardization.

### Identification of m5C-Related lncRNAs

Based on the previous studies, m5C regulators were obtained, including YBX1, ALYREF, DNMT1, NSUN4, TRDMT1, TET2, NSUN7, NSUN6, NSUN5, NSUN3, NSUN2, DNMT3A, and DNMT3B (Nombela et al., 2021). Then, Pearson’s correlation analysis was performed to identify the m5C-related lncRNAs with the criteria |R|>0.4 and *p* < 0.001. Next, univariate Cox regression analysis was performed to screen the lncRNAs remarkably associated with patient prognosis (*p* < 0.05).

### Construction of the Prognosis Model

All the patients were randomly divided into training and validation cohorts according to the 1:1 ratio. Based on the prognosis-related lncRNAs identified by the univariate Cox regression analysis, LASSO regression and multivariate Cox regression analysis were then performed for model construction (Tibshirani, 1997). The predictive model was constructed with the formula of “Riskscore = ∑exp(lncRNAs) ∗ β”, in which “β” represents the coefficient of each lncRNAs from Cox analysis. The Kaplan–Meier survival curve and receiver operating characteristic (ROC) curve were used to evaluate the prediction efficiency of our prognosis model.

### Nomogram and Calibration Curve

A nomogram plot was constructed with the combination of risk score and clinical features for a better application in the clinic (Pan X. et al., 2021). Calibration curves were used to compare the fit between the nomogram-predicted survival and actual survival (Van Calster et al., 2016).

### Pathway Enrichment Analysis

To further explore the underlying biological differences between the high- and low-risk groups, pathway enrichment analysis was performed based on the Gene Set Enrichment Analysis (GSEA) algorithm, which is a useful tool for genomic study (Subramanian et al., 2005). The GSEA algorithm can evaluate the microarray data at the gene set level, and the gene set used was defined based on the previous biological knowledge. The reference enrichment file was the Hallmark gene set.

### Immune Infiltration Analysis

The tumor microenvironment is a key factor affecting the progression of cancer. The CIBERSORT algorithm was used to perform immune infiltration analysis, which could quantify the relative proportions of 22 types of infiltrating immune cells (Chen et al., 2018).

### Immunotherapy and Chemotherapy Sensitivity Analysis

Tumor Immune Dysfunction and Exclusion (TIDE) and submap algorithm were used to evaluate the difference in the immunotherapy response rate between the high- and low-risk groups (Fu et al., 2020). The Genomics of Drug Sensitivity in Cancer (GDSC) database was used to assess the chemotherapy sensitivity, which was performed using the pRRophetic package (Yang et al., 2013).

### Statistical Analysis

R software v4.0.0 and SPSS 13.0 were used for statistical analysis. *p*-value was two-sided and less than 0.05 was considered as statistically significant. Student’s t-test and Wilcoxon rank-sum test were used for the continuous variables.

## Results

### Identification of m5C-Related lncRNAs

**FIGURE 1:**
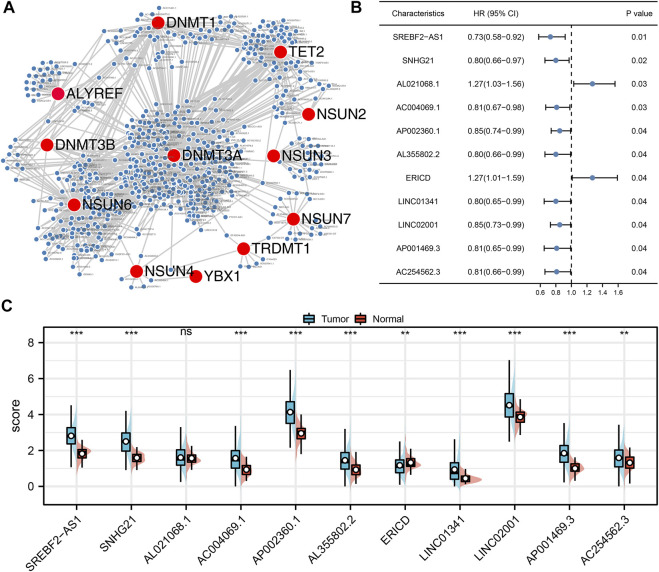
Identification of the m5C-related lncRNAs. **(A)** Co-expression network of m5C genes and the m5C-related lncRNAs; **(B)** Univariate Cox regression analysis was performed to identify the prognosis-related lncRNAs; **(C)** Expression level of SREBF2-AS1, SNHG21, AL021068.1, AC004069.1, AP002360.1, AL355802.2, ERICD, LINC01341, LINC02001, AP001469.3, and AC254562.3 in normal and tumor tissue.

The baseline information of enrolled patients is shown in Table 1. With the criterion of |R|>0.4 and p < 0.001, a total of 614 m5C-related lncRNAs were identified (Figure 1A). After that, the univariate Cox analysis was performed to screen the prognosis-related lncRNAs. The results showed that the lncRNAs SREBF2-AS1, SNHG21, AL021068.1, AC004069.1, AP002360.1, AL355802.2, ERICD, LINC01341, LINC02001, AP001469.3, and AC254562.3 were significantly associated with patient prognosis (Figure 1B). The expression difference of these prognosis-related lncRNAs is shown in Figure 1C.

**TABLE 1:** Baseline data of the enrolled patients.

Features		Numbers	Proportion (%)
Age	≤65	190	37.9
	>65	302	60.3
	Unknown	9	1.8
Gender	Female	130	25.9
	Male	371	74.1
Stage	Stage I	244	48.7
	Stage II	162	32.3
	Stage III	84	16.8
	Stage IV	7	1.4
	Unknown	4	0.8
T classification	T1	114	22.8
	T2	293	58.5
	T3	71	14.2
	T4	23	4.6
M classification	M0	411	82.0
	M1	7	1.4
	Unknown	83	16.6
N classification	N0	319	63.7
	N1	131	26.1
	N2	40	7.9
	N3	5	0.9
	Unknown	6	1.2

### Prognosis Model Construction and Validation

**FIGURE 2:**
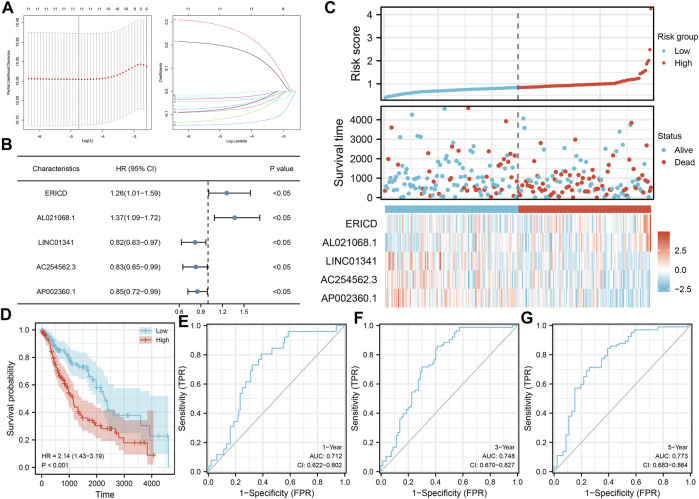
Construction of the prognosis model. **(A)** LASSO regression analysis was used for dimension reduction; **(B)** ERICD, AL021068.1, LINC01341, AC254562.3, and AP002360.1 were identified for prognosis model construction; **(C)** Overview of our model in the training cohort; **(D)** Kaplan–Meier survival curve was performed to indicate the prognosis difference between high- and low-risk groups; **(E–G)** ROC curve of 1-, 3-, and 5-years.

**FIGURE 3:**
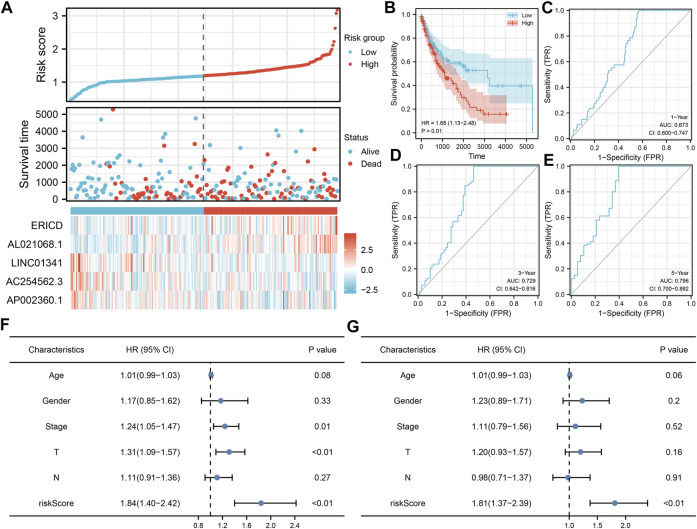
Validation of the prognosis model. **(A)** Overview of our model in the validation cohort; **(B–E)** Kaplan–Meier survival and ROC curves in the validation cohorts; **(F–G)** Univariate and multivariate analysis of our model.

Based on these prognosis-related lncRNAs, LASSO regression was then performed for dimensionality reduction (Figure 2A). Multivariate Cox analysis was then performed for model construction, and the lncRNAs ERICD, AL021068.1, LINC01341, AC254562.3, and AP002360.1 were identified (Figure 2B). In the training group, a higher proportion of dead cases was observed in the high-risk groups (Figure 2C).The Kaplan–Meier survival curve showed that the patients in the high-risk group tend to have a worse prognosis compared with those in the low-risk group (Figure 2D). The ROC curve indicated a great prediction efficiency of our model in 1-, 3-, and 5-years (Figures 2E–G, 1-year AUC = 0.712). In the validation cohorts, the same trend was also observed (Figure 3A). The Kaplan–Meier survival curve indicated that the high-risk patients might have a shorter overall survival (OS) than the low-risk patients (Figure 3B). Also, the ROC curve showed a good prediction efficiency in patients prognosis (Figures 3C–E, 1-year AUC = 0.673, 3-years AUC = 0.729, and 5-years AUC = 0.796). Univariate and multivariate analysis showed that our model was a prognosis biomarker independent of other clinical features (Figures 3F,G, univariate, HR = 1.84, p < 0.01; multivariate, HR = 1.81, p < 0.01).

### Nomogram and Clinical Correlation

**FIGURE 4:**
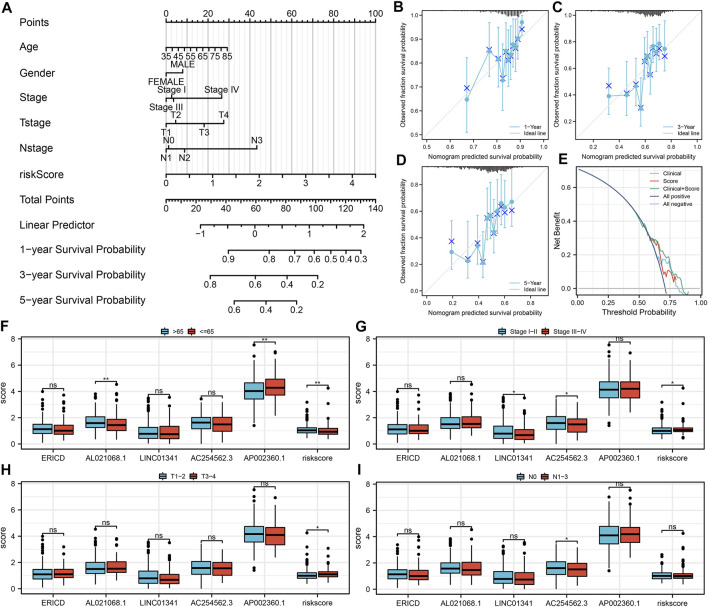
Nomogram and clinical correlation analysis. **(A)** Nomogram was constructed by combining the risk score and clinical features; **(B–D)** Calibration curves of the nomogram; **(E)** DCA curve of the nomogram; **(F)** Expression of model lncRNAs and risk score in different age groups; **(G)** Expression of model lncRNAs and risk score in different clinical stage groups; **(H)** Expression of model lncRNAs and risk score in different T classifications groups; **(I)** Expression of model lncRNAs and risk score in different N classifications groups.

Next, a nomogram plot was established by combining the risk score and clinical features (Figure 4A). The calibration curves demonstrated a high goodness-of-fit between the nomogram-predicted survival and actual survival (Figures 4B–D). The DCA curve showed that the clinical features could improve the prediction efficiency of our model (Figure 4E). Clinical correlation analysis showed that the risk score was correlated with worse clinical features, including age, clinical-stage, and T classification (Figures 4F–I); the older patients might have a higher AL021068.1 but a lower AP002360.1 expression (Figure 4F); the progressive clinical-stage patients might have a lower LINC01341 and AC254562.3 expression (Figure 4G); the progressive N classification might have a lower AC254562.3 expression (Figure 4I).

### Pathway Enrichment and Immune Infiltration Analysis

**FIGURE 5:**
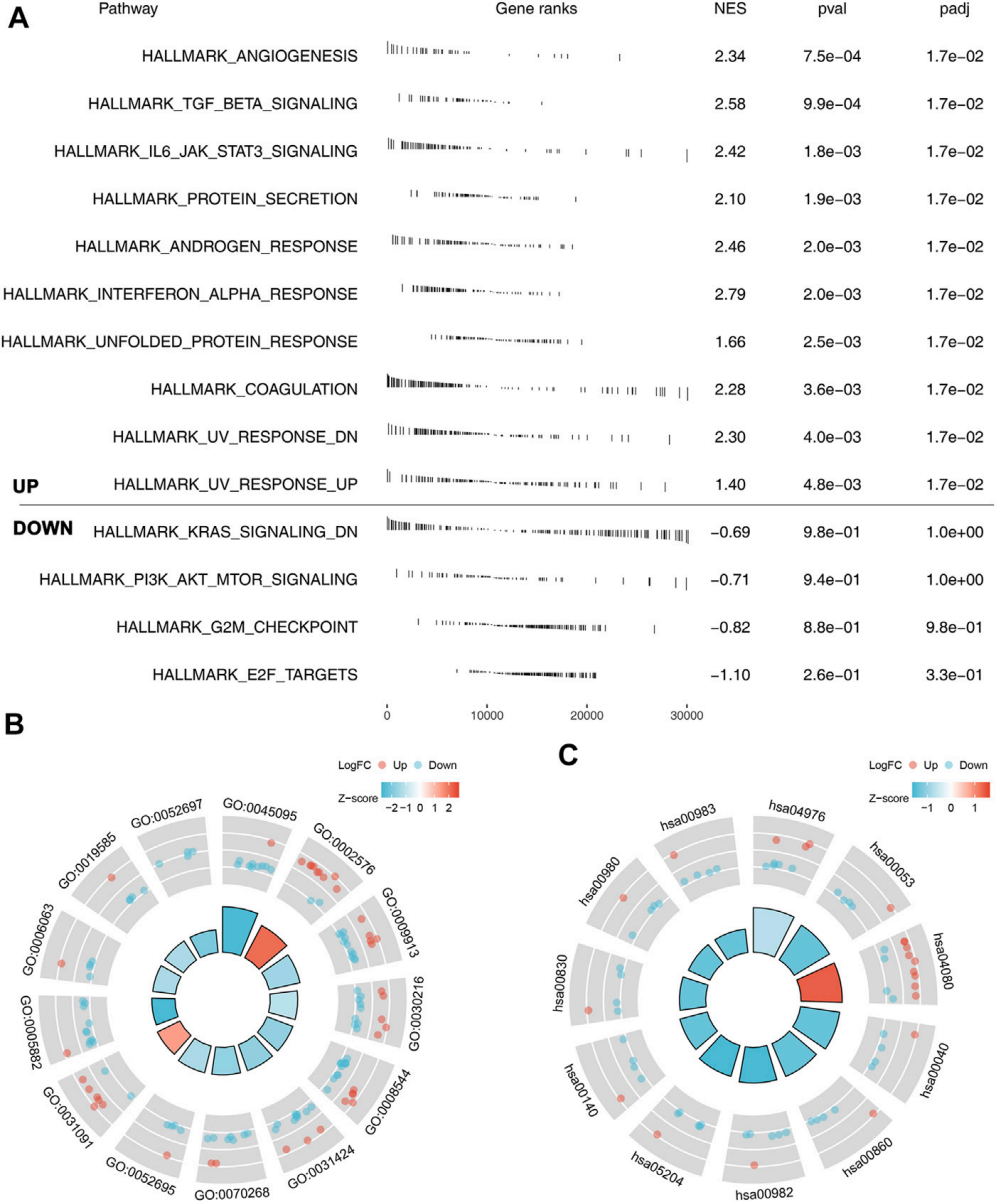
Pathway enrichment analysis of our model. **(A)** GSEA analysis of the model based on the Hallmark gene set; **(B)** GO analysis of our model; **(C)** KEGG analysis of our model.

**FIGURE 6:**
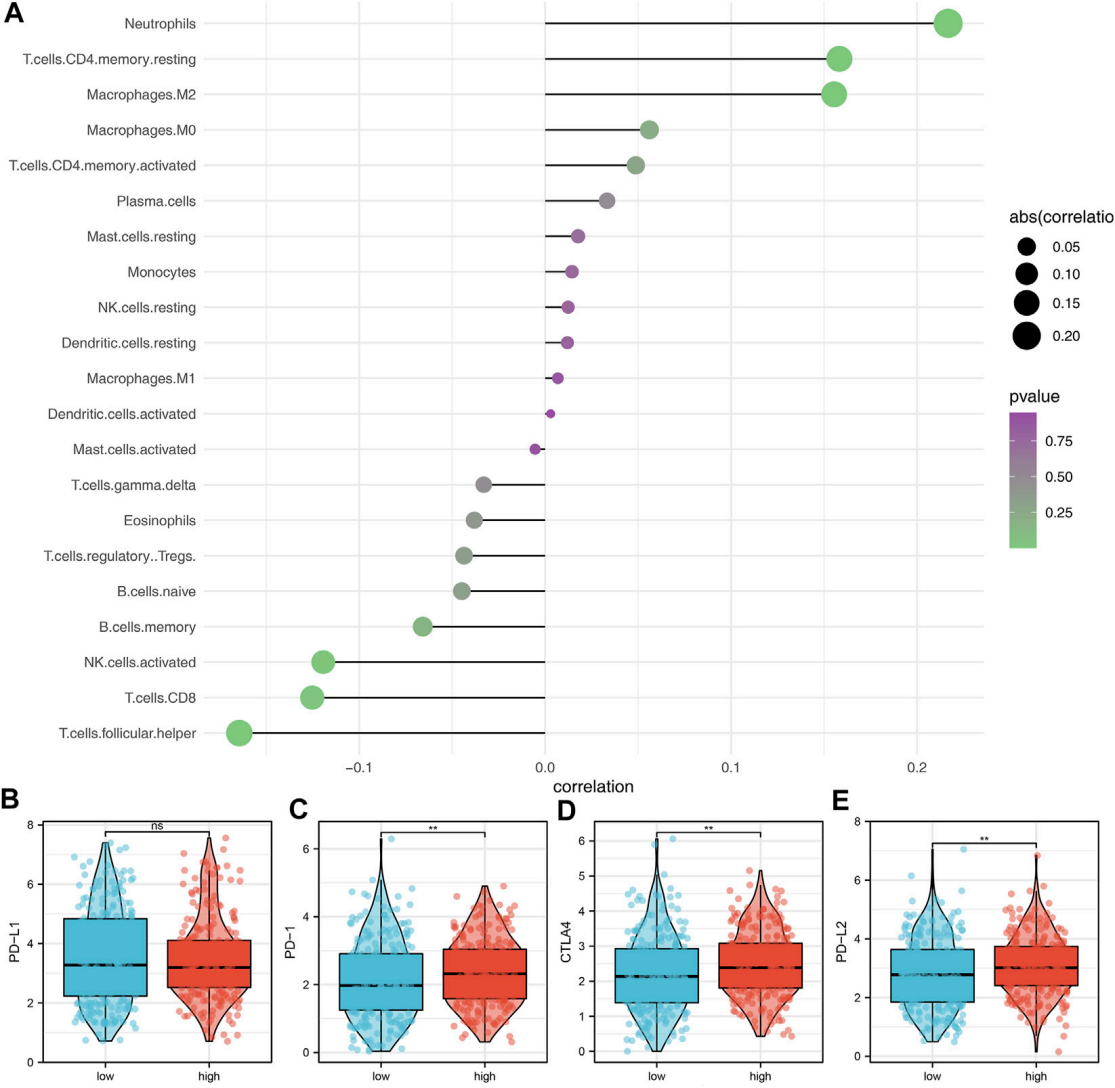
Immune infiltration analysis. **(A)** Immune infiltration analysis was performed using the CIBERSORT algorithm; **(B–E)** Expression of level of PD-L1, PD-1, CTLA4, and PD-L2 in the high- and low-risk groups.

The underlying biological pathway and immune microenvironment difference might lead to prognosis difference between the high- and low-risk groups. Pathway enrichment analysis showed that in the high-risk group, the pathways of angiogenesis, TGF-β signaling, IL6-JAK-STAT3 signaling, protein secretion, androgen response, interferon-α response, and unfolded protein response were significantly enriched (Figure 5A). The GO and KEGG analysis showed that the terms of platelet degranulation (GO:0002576), platelet alpha granule (GO:0031091), and neuroactive ligand-receptor interaction (hsa04080) were significantly upregulated in high-risk patients (Figures 5B,C). Immune infiltration analysis indicated that the risk score was positively correlated with neutrophils, resting CD4+ memory T cells, and M2 macrophages, yet negatively correlated with follicular helper T cells, CD8+ T cells, and activated NK cells (Figure 6A). We further explored the differences in key immune checkpoints between the high- and low-risk patients, including PD-L1, PD-1, CTLA4, and PD-L2. The results showed that PD-1, CTLA4, and PD-L2 were significantly upregulated in the high-risk patients, but not in PD-L1 (Figures 6B–E).

### Immunotherapy and Chemotherapy Sensitivity

**FIGURE 7:**
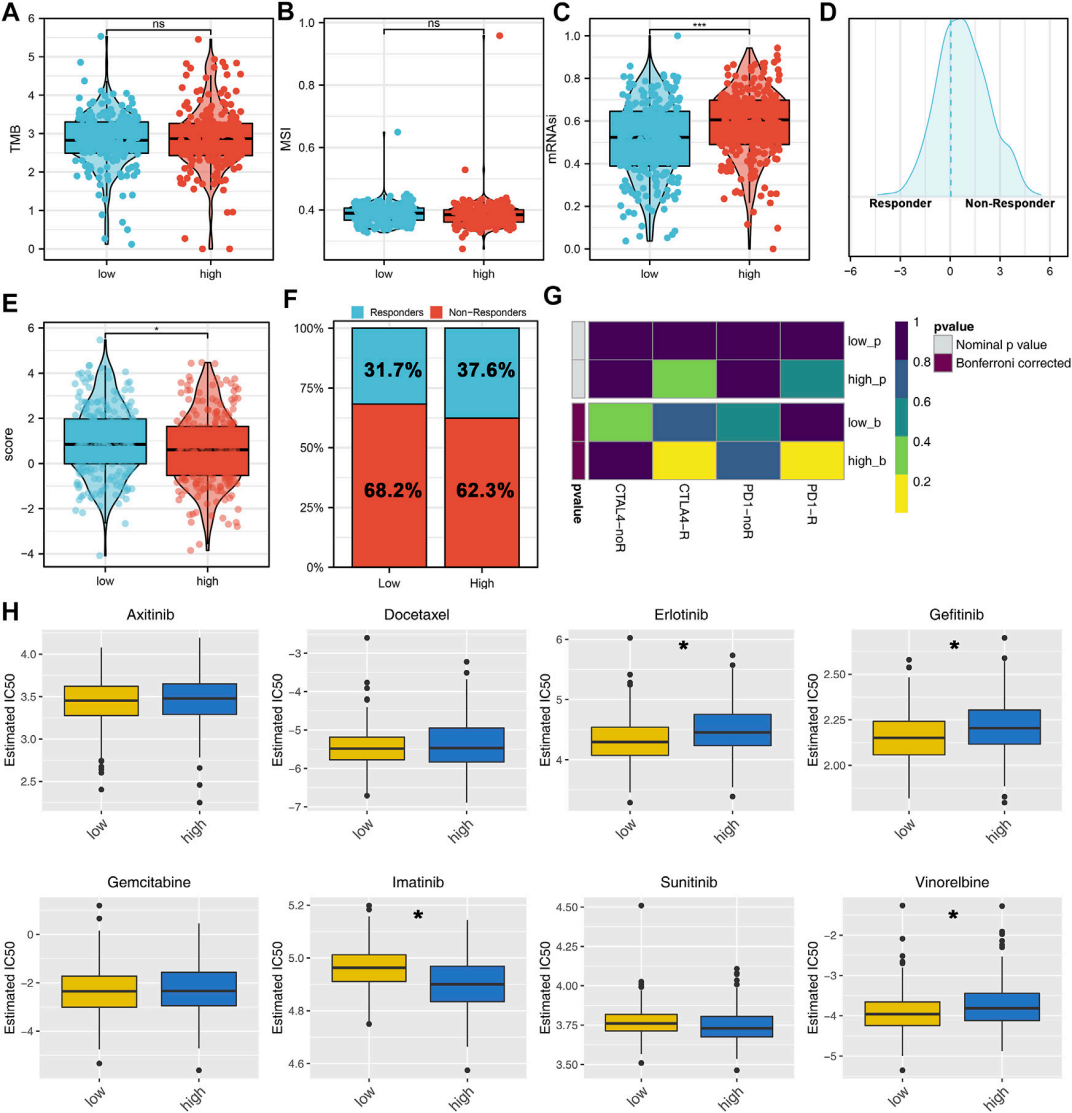
Immunotherapy and drug sensitivity analysis. **(A–C)** TMB, MSI, and mRNAsi differences between high- and low-risk patients; **(D)** TIDE analysis was performed to evaluate the immunotherapy response of patients, among which TIDE >0 was defined as non-responders and <0 was defined as responders; **(E)** Difference of TIDE score between the high- and low-risk groups; **(F)** High-risk group had a higher percentage of immunotherapy responders; **(G)** Submap algorithm was performed to explore the specific immunotherapy options; **(H)** Chemosensitivity difference between the high- and low-risk patients.

Next, the genomic TMB, MSI, and mRNAsi differences were explored between the high- and low-risk patients. No significant differences were observed in TMB and MSI between high- and low-risk patients (Figures 7A,B). However, we found a higher mRNAsi in the high-risk patients (Figure 7C). Immunotherapy is an important therapy option for lung cancer. Therefore, we explored the immunotherapy response rate between high- and low-risk patients. According to the TIDE analysis, the TIDE score <0 was defined as responders (Figure 7D). Meanwhile, we found that the high-risk patients have a lower TIDE score and a higher proportion of responders (Figures 7E,F). Submap analysis showed that the high-risk patients might be more sensitive to both PD-1 and CTLA4 therapy (Figure 7G). Furthermore, we explored the chemotherapy sensitivity differences of common drugs in lung cancer between high- and low-risk patients. The results showed that the high-risk patients might be more sensitive to imatinib, yet resistant to erlotinib, gefitinib, and vinorelbine (Figure 7H).

## Discussion

Lung cancer is a common tumor all over the world (Brody, 2014). Along with the change of lifestyle, the morbidity of lung cancer increased gradually (Brody, 2014). Though surgery could lead to a good prognosis for the most lung cancer patients, however, for those advanced patients, the prognosis is still unsatisfactory.

In our study, we identified the m5C-related lncRNAs based on the TCGA data. Then, a prognosis model based on ERCID, AL021068.1, LINC01341, AC254562.3, and AP002360.1 was established, which had good prediction efficiency in both the training and validation cohorts. Univariate and multivariate analysis showed that the risk score is an independent prognosis factor. A nomogram plot was then constructed for a better clinical application. Pathway enrichment and immune infiltration analysis were performed to explore the underlying biological differences between high- and low-risk patients. Moreover, we found that the high-risk patients might be more sensitive to immunotherapy. Meanwhile, we also found a significant sensitivity difference of imatinib, erlotinib, gefitinib, and vinorelbine between the high- and low-risk patients.

Our study established a prognosis model consisting of five m5C-related lncRNAs, ERCID, AL021068.1, LINC01341, AC254562.3, and AP002360.1. In LUSC, prognosis models based on different molecules have been established. Pan et al. (2021b) revealed that the prognosis model based on NSUN3 and NUSN4 could indicate patient survival. However, the AUC of 3-year ROC curves was only 0.561 and 0.629 in the training and validation cohorts, respectively. Pan et al. (2021a) also explored the role of m5C-related lncRNAs in LUAD. They found that the expression level of 14 m5C-related lncRNAs can robustly predict patient prognosis. To the best of our knowledge, this is the first study focused on the role of m5C-related lncRNAs in LUSC. Our model can not only effectively predict the prognosis of LUSC patients but also indicate the immunotherapy sensibility of patients, which has the potential for clinical applications.

Epigenetic regulation plays an important role in the tumor progression process (Garcia-Martinez et al., 2021). Recently, m5C modifications have gradually attracted the attention of researchers. Wang et al. found that the PKM2 m5C modification regulated by the HIF-1α/ALYREF/PKM2 axis could facilitate bladder cancer progression through the glucose metabolism manner (Wang et al., 2021). Yang et al. (2021) revealed that the RNA methyltransferase NSUN6 could hamper pancreatic cancer development by regulating cell proliferation. Su et al. (2021) demonstrated that NSUN2-mediated m5C modification could facilitate esophageal squamous cell carcinoma development through LIN28B-dependent GRB2 mRNA stabilization. Moreover, Ma et al. (2022) established a database with a comprehensive collection and annotation of RNA 5-methylcytosine, named m5C-Atlas. The modification of m5C in diseases is gradually attracting attention. Our study identified the lncRNAs associated with m5C genes, which might be the underlying prognosis and therapeutic targets of LUSC.

Pathway enrichment analysis showed that the pathways of angiogenesis, TGF-β signaling, IL6-JAK-STAT3 signaling, protein secretion, androgen response, interferon-α response, and unfolded protein response were activated in high-risk patients. Angiogenesis is essential for tumor progression and distant metastasis (Viallard and Larrivée, 2017). The pro-angiogenic secreted by tumor cells contribute to the formation of a complex and immature vessel network, leading to a poor perfusion and a hypoxic microenvironment in cancer (Viallard and Larrivée, 2017). Also, in lung cancer, Wang et al. found that the circular RNA hsa_circ_0008305 (circPTK2) could hamper the TGF-β-induced epithelial–mesenchymal transition and metastasis by controlling TIF1γ in lung cancer (Wang et al., 2018). Shen et al. (2019) demonstrated that the inhibition of ATM could decrease the metastatic potential of cisplatin-resistant lung cancer cells through the JAK/STAT3/PD-L1 pathway. Meanwhile, lanatoside C could induce tumor proliferation and G2/M cell cycle arrest by attenuating the JAK/STAT signaling pathway (Reddy et al., 2019).

Immune infiltration analysis showed that the risk score was positively correlated with neutrophils, resting CD4+ memory T cells, and M2 macrophages, yet negatively correlated with follicular helper T cells, CD8+ T cells and activated NK cells. Faget et al. (2017) found that the neutrophils and snail could orchestrate the establishment of a pro-tumor microenvironment in lung cancer. Also, Ancey et al. (2021) found that the GLUT1 expressed in tumor-associated neutrophils could promote lung cancer growth and resistance to radiotherapy. M2 macrophages generally act as a cancer-promoting factor in solid tumors, including lung cancer (Locati et al., 2020). Activated NK cells and CD8+ T cells can kill cancer cells, which are important for cancer immunotherapy (Myers and Miller, 2021). Meanwhile, some novel therapy option based on these has achieved encouraging results (Lin et al., 2020). Immunotherapy and chemotherapy are two important therapy choices for lung cancer patients (Steven et al., 2016). Our results showed that the high-risk patients might be more sensitive to immunotherapy, imatinib, yet resistant to erlotinib, gefitinib, and vinorelbine, indicating our model has the underlying ability to guide clinical treatment.

Some limitations should be noticed. First, the population included in our study was predominantly Western populations. Given the underlying biological difference between different races, it would bring race bias to the application of our model in other populations. Second, some clinical features of patients are still hard to obtain. For example, the M classification of most patients is unknown, which was removed from our analysis. Therefore, if most clinical features can be downloaded, our conclusion might be more stable.

## Conclusion

In all, our study comprehensively explored the role of m5C-related lncRNAs in LUSC. Our results indicated that the prognosis model based on ERICD, AL021068.1, LINC01341, AC254562.3, and AP002360.1 might be an effective tool that could robustly predict LUSC patient prognosis and immunotherapy sensibility, which had the potential for clinical guidance.

## Data Availability

Publicly available datasets were analyzed in this study. These data can be found here: https://portal.gdc.cancer.gov/.
